# Why do patients go off track? Examining potential influencing factors for being at risk of psychotherapy treatment failure

**DOI:** 10.1007/s11136-020-02664-6

**Published:** 2020-10-21

**Authors:** Viola N. L. S. Schilling, Dirk Zimmermann, Julian A. Rubel, Kaitlyn S. Boyle, Wolfgang Lutz

**Affiliations:** 1 Department of Psychology, Clinical Psychology and Psychotherapy, University of Trier, 54286 Trier, Germany; 2 Justus-Liebig-University Giessen, Giessen, Germany

**Keywords:** Psychotherapy, Routine outcome monitoring, Feedback, Clinical support tools

## Abstract

**Background:**

Routine outcome monitoring can support clinicians to detect patients who deteriorate [not-on-track (NOT)] early in psychotherapy. Implemented Clinical Support Tools can direct clinicians’ attention towards potential obstacles to a positive treatment outcome and provide suggestions for suitable interventions. However, few studies have compared NOT patients to patients showing expected progress [on-track (OT)] regarding such obstacles. This study aimed to identify domains that have predictive value for NOT trajectories and to compare OT and NOT patients regarding these domains and the items of the underlying scales.

**Methods:**

During treatment, 413 outpatients filled in the Hopkins-Symptom-Checklist-11 (depressive and anxious symptom distress) before every therapy session as a routine outcome measure. Further, the Assessment for Signal Clients, Affective Style Questionnaire, and Outcome Questionnaire-30 were applied every fifth session. These questionnaires measure the following domains, which were investigated as potential obstacles to treatment success: risk/suicidality, therapeutic alliance, motivation, social support and life events, as well as emotion regulation. Two groups (OT and NOT patients) were formed by defining a cut-off (failure boundary) as the 90% confidence interval (upper bound) of the respective patients’ expected recovery curves. In order to differentiate group membership based on the respective problem areas, multilevel logistic regression analyses were performed. Further, OT and NOT patients were compared with regard to the domains’ and items’ cut-offs by performing Pearson chi-square tests and independent samples *t*-tests.

**Results:**

The life events and motivation scale as well as the risk/suicidality scale proved to be significant predictors of being not-on-track. NOT patients also crossed the cut-off significantly more often on the domains risk/suicidality, social support, and life events. For both OT and NOT patients, the emotion regulation domain’s cut-off was most commonly exceeded.

**Conclusion:**

Life events, motivation, and risk/suicidality seem to be directly linked to treatment failure and should be further investigated for the use in clinical support tools.

**Electronic supplementary material:**

The online version of this article (10.1007/s11136-020-02664-6) contains supplementary material, which is available to authorized users.

## Introduction

Routine outcome monitoring (ROM) and feedback to therapists and patients during treatment can be an effective and cost-efficient method to improve patient outcomes in psychotherapy [1–4]. In the past two decades, many institutions all over the world have therefore started implementing ROM in their services systems [5–8]. Feedback from outcome measures can support clinicians to detect patients who deteriorate early in the treatment process and enable clinicians to adapt their treatment strategy as needed. Implementing progress feedback seems particularly important considering that studies have shown that statistical methods outperform clinicians in predicting treatment failure and that the accuracy of predictions can be improved by approximately 13% when statistical algorithms are applied [9, 10].

Since feedback on outcomes does not necessarily provide any information on how to adjust the treatment strategy, other sources of information need to be consulted. Adding so-called clinical support tools (CST) to feedback can help to provide such information [11]. CSTs can be defined as problem-solving tools, which alert the clinician to potential obstacles to a positive treatment outcome and provide suggestions for possible interventions. The domains upon which CSTs are based should be relevant to change processes and optimally be applicable to many patients with different psychopathological diagnoses. Asay and Lambert [12] named four general factors particularly related to change. Based on empirical findings, the authors argue that 40% of recovery can be attributed to (1) client variables and extratherapeutic factors. Further, 30% of improvement can be ascribed to (2) therapeutic relationship factors, while (3) hope and expectancy factors are as important as (4) models and technique factors, each accounting for 15% of recovery.

Lambert et al. [13] developed the Assessment for Signal Clients (ASC), a self-report questionnaire that assesses three of these four factors. Extratherapeutic factors are measured by two subscales, namely social support and life events. Relationship factors are assessed by a therapeutic alliance scale and hope and expectancy factors are operationalized by a motivation scale. Clinical cut-off scores are provided for each scale. Further, item cut-offs help the clinician to determine which of the scale’s items are most critical. Therapists are also provided with a decision tree [13], guiding them through the four scales (1) therapeutic alliance, (2) motivation, (3) social support, and (4) stressful life events hierarchically. Further, therapists are guided to reevaluate the diagnosis, need for medication, and the treatment method.

Research has shown that feedback on patient progress is especially effective (i.e., improves treatment outcome) for patients whose symptom distress develops negatively over the course of treatment (negative change trajectory), so-called not-on-track (NOT) patients [14]. A recent meta-analysis by Lambert et al. [3] reported a weighted effect size of feedback versus treatment as usual (TAU) of .33 for NOT patients (small effect). To further enhance these effects, CSTs are added to feedback [3, 14]. In a meta-analysis by Shimokawa et al. [14], the mean effect size for the combination of feedback and CSTs versus TAU reached *g* = .70 (medium to large effect) for NOT patients, while Lambert et al. [3] found a lower but still considerable mean effect size of *g* = .49 (small to medium effect).

Research that particularly focuses on CST domains (the categories or sections that structure the different tools, for instance, therapy motivation or social support) is relatively scarce. Two studies have investigated ASC data to find out more about potential obstacles to a positive outcome. White et al. [15] examined ASC data from 107 NOT patients from a hospital-based outpatient clinic. About 58% of patients presented with enough problems to exceed a clinical cut-off on at least one of the four ASC scales. In other words, for more than 40% of NOT patients, it was not possible to identify a potential obstacle to positive treatment outcome. This could indicate that more domains should be examined to be able to identify underlying obstacles to successful treatment for more patients. Probst et al. [16] evaluated the importance of the ASC scales in a sample of patients showing extreme deviations from their statistically generated expected recovery curves. The life events and social support domains were associated with extreme negative deviations. The authors concluded that prioritizing extratherapeutic factors in the decision tree might help to prevent treatment failure.

Building on findings by White et al. [15], further domains beyond those assessed by the ASC may be relevant to patient deterioration and important to consider when implementing CSTs. Emotion regulation as well as risk behavior and suicidality could be worthy candidates when implementing CSTs. Emotion regulation is a process that has previously been associated with the development and maintenance of clinical disorders [17], but it has not been implemented in CSTs so far. It comprises different affective styles that influence the quality, intensity, timing, and duration of emotions [18]. Three emotion regulation strategies that have been consistently found in the literature are tolerating, adjusting, and concealing emotions [19–21]. An instrument that assesses individual differences in emotion regulation is the Affective Style Questionnaire (ASQ), developed by Hofman and Kashdan [21]. In contrast to emotion regulation, risk behavior like drinking or substance abuse as well as suicidality is assessed in other systems, but these factors are not usually implemented as an individual domain. However, they can have a major impact on the course of therapy and clinicians may profit from more information on these topics [22]. This gap could be closed by implementing an extra domain in feedback systems that covers risk and suicidality.

Although many studies have focused on the question of whether feedback is effective [3, 14, 23], questions regarding the implementation and explanatory power of domains selected as the basis of CSTs remain unanswered. To date, few studies have been conducted that compare OT and NOT patients regarding the domains and individual items upon which CSTs are based. A comprehensive picture on the factors that lead to treatment failure, however, is necessary in order to prevent deterioration in therapy by means of feedback and CSTs. The current study therefore aims to evaluate CST domains that are associated with treatment failure. More specifically, we strive to find out more about the difference between OT and NOT patients regarding these domains and the individual items of the underlying scales. This is important in order to not only be able to provide feedback that the treatment strategy should be adjusted, but more specifically to indicate which strategies or interventions can be used to optimize treatment outcomes. This knowledge can be used to support the continued development of ROM systems.

The current study aims to investigate the following research questions:

1. Which domains have predictive value for NOT trajectories?
2. Do OT and NOT patients differ regarding how often they surpass the domain cut-offs?
3. Do OT and NOT patients score differently on the individual items assessing the potential obstacles to a positive treatment outcome?

## Methods

### Sample

**Fig. 1:**
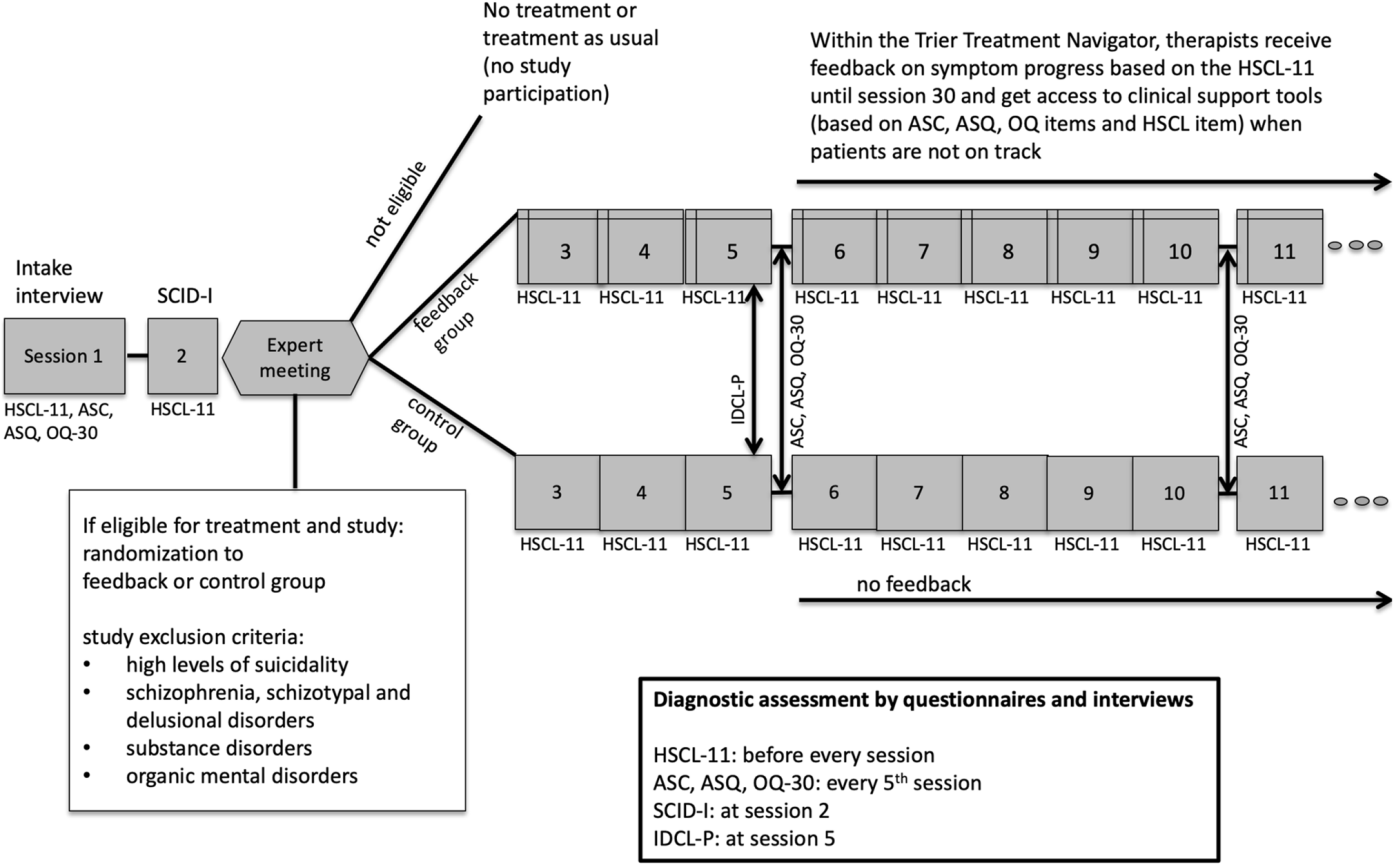
Flow diagram depicting the procedure within the RCT. *IDCL-P* International Diagnostic Checklist for Personality Disorders, *ASC* Assessment for Signal Clients, *ASQ* Affective Style Questionnaire, *HSCL-11* Hopkins Symptom Checklist-11, *OQ-30* Outcome Questionnaire-30, *SCID-I* Structured Clinical Interview for DSM-IV

The analyses were based on 413 patients receiving cognitive-behavioral therapy (CBT) delivered by 65 therapists within a randomized controlled trial (RCT [7, 24]) examining the effectiveness of ROM in an outpatient center in Western Germany. When sample selection for this study took place, the RCT was still ongoing. Treatments were conducted by CBT trainees, who had at least 1.5 years of clinical experience. Patients were enrolled in the program using the following procedures (Fig. 1). All patients attended an intake interview conducted by intensively trained independent clinicians and completed the Hopkins Symptom Checklist-11 (HSCL-11 [25]), the Assessment for Signal Clients (ASC [13]), the Affective Style Questionnaire (ASQ [21, 26]), and the Outcome Questionnaire (OQ-30 [27]). During the second visit, patients went through a diagnostic interview, in which past and current psychological disorders were assessed by the German version of the *Structured Clinical Interview for DSM-IV* (SCID-I [28]). After the second visit, an expert panel composed of four senior clinicians evaluated each patient for program eligibility. The following exclusion criteria were applied: high levels of suicidality, schizophrenia, schizotypal and delusional disorders, substance disorders, and organic mental disorders. Eligible patients were then randomized to either the “feedback group” or the “control group” using a computerized algorithm (the ratio feedback:control was 2:1). Of the *n* = 413 patients, *n* = 157 patients were included in the control group and *n* = 256 patients in the feedback group. Patients filled in the HSCL-11 before every therapy session as a routine outcome measure. Further, the ASC, ASQ, and OQ-30 were applied every fifth session. A description of these measures is provided below. Personality disorders were assessed by means of the *International Diagnostic Checklist for Personality Disorders* (IDCL-P [29]) in session five. If patients attended less than six sessions, they were excluded from the analysis as categorization into OT and NOT patients starts at session six. To evaluate therapists’ attitudes towards feedback and experiences with the feedback system, therapists filled in evaluations after completion of treatment with their individual patients. Further, user statistics for each therapist were recorded to evaluate the frequency of feedback use and time spend within the system for each individual case. All sessions were video-taped and a selection of the videos was rated regarding competence and adherence [30] by trained master-level and post-graduate-independent raters. A detailed description of the assessment of these control variables can be found in [7]. The study was approved by the local Ethics Committee of the University of Trier.

### Instruments

#### Hopkins Symptom Checklist-11 (HSCL-11)

The HSCL-11 [25], a modified version of the Symptom Checklist-90-R [31] consisting of 11 items assessing depressive and anxiety symptoms, is scored on a 4-point Likert scale ranging from *not at all (0)* to *very much (3)*. In our sample, the reliability was *α* = .87 at session six. The HSCL-11 was administered on a touch screen prior to every session as a routine outcome measurement of symptom distress. As part of a comprehensive feedback system (“Trier Treatment Navigator” section), the HSCL-11 was used to track change and distinguish patients at risk from patients not at risk of treatment failure. Along with two OQ-30 items, the HSCL item “In the past seven days, how much were you distressed by thoughts of ending your life” was further used as an indicator for suicidal problems within the risk/suicidality domain [24]. The cut-off score was set at ≥ 2 (*quite a lot*).

#### Assessment for Signal Clients (ASC)

The ASC [13] is a 40-item self-report questionnaire that assesses four areas: therapeutic alliance (TA—the emotional bond between the patient and therapist as well as agreement on the goals and tasks of therapy), motivation (MO—patient motivation to work on problems and expectancy that therapy is helpful), social support (SS—whether patients have people they can talk to about their problems and who support them), and life events (LE—the amount of distressing negative life events). TA and SS subscales are comprised of eleven items each, while the subscales MO and LE each consist of nine items. The items are summed up to subscales. In order to simplify interpretation, reversed items were recoded so that high values indicate high functioning. In our sample, the Cronbach’s alphas at session six were TA: *α* = .84; SS: *α* = .81; MO: *α* = .71, and LE: *α* = .74. The ASC cut-offs for warning signals were based on previously reported scores for the four subscales (alliance ≤ 39, social support ≤ 23, motivation ≤ 32, and critical life events ≤ 23; [32]). The cut-off for the individual items (after recoding reversed items) was ≤ 2. Patients were assessed using the ASC via paper–pencil questionnaires every fifth session.

#### Affective Style Questionnaire (ASQ)

The ASQ [21, 26] assesses three broad emotion regulation styles with 20 items on a 5-point Likert scale (*not true of me at all* to *extremely true of me*). *Concealing* (8 items) refers to an emotion regulation style that is characterized by the avoidance or suppression of emotions. *Tolerating* (5 items) refers to a non-defensive and accepting attitude towards negative emotions, whereas *adjusting* (7 items) refers to the management and reappraisal of emotions in order to improve well-being. For better interpretation, items from the concealing scale were recoded. In our sample, the Cronbach’s alphas at session six were the following: concealing: *α* = 0.88; adjusting: *α* = .82; tolerating: *α* = .76. The cut-offs for the ASQ were based on an archival dataset of *N* = 1150 outpatients at session five [24]. To calculate the cut-off scores, 1 standard deviation was subtracted from the mean (concealing = 3.01–0.74, tolerating = 2.97–0.68, and adjusting = 2.45–0.75). The cut-off scores for the individual items were ≤ 2 for tolerating, adjusting, and concealing (after recoding). Patients filled in the ASQ via paper–pencil questionnaires every fifth session.

#### Outcome Questionnaire-30

The 30-item self-report instrument is a short version of the OQ-45 [33], which evaluates treatment outcome on the dimensions subjective discomfort, interpersonal relationships, and social role performance. The questionnaire demonstrates adequate psychometric properties [34]. In this outpatient sample, internal consistency was found to be excellent at session six (*α* = .92). Although all patients filled in the entire OQ-30 every fifth session, for the purpose of the analyses, we only made use of two of the instrument’s items (item 5: “I have thoughts of ending my life” and item 18: “I feel annoyed by people who criticize my drinking (or drug use)).” These items are the basis of the risk/suicidality tool in the Trier Treatment Navigator. As soon as one of the items reached a score ≥ 3 (often), therapists received a warning signal within the risk/suicidality tool. The OQ-30 was administered every fifth session via paper–pencil questionnaires. Online resource 1 summarizes the domain and item cut-offs of the implemented questionnaires and scales.

#### Trier Treatment Navigator (TTN)

The TTN [24] is a comprehensive feedback system that supports clinicians in the decision-making process before and during treatment. It consists of two parts: (1) personalized pre-treatment recommendations that provide information about the estimated drop-out risk and the predicted optimal treatment strategy for the first ten sessions and (2) personalized adaptive recommendations that support clinicians during the therapy process to identify at-risk patients by means of a dynamic risk index and the CST to support adjustment of the treatment strategy if necessary. An illustration of the different components of the TTN can be found in online resource 2.

In order to determine OT and NOT patients during treatment, a dynamic failure boundary is calculated and updated every session (starting at session six and ending at session 30), taking into account the change from intake up to the current session. The calculation is based on an archival dataset of *n* = 1234 outpatients and relies on the nearest neighbors approach [35]. Only patients with a positive slope, that is, a successful course of treatment, are selected as “nearest neighbors”. To model treatment progress for each individual patient based on their nearest neighbors, impairment assessed by the HSCL-11 was regressed on the logarithmized time variable (i.e., session number), the total number of sessions per patient as well as a cross-level interaction between the two. The failure boundary is defined as the upper limit of the 90% confidence interval. Each time the patient surpasses the failure boundary, the system generates a warning signal for the therapist, indicating that the patient’s progress is not as expected and that an adaptation of the treatment strategy might be necessary. In order to get back on track, impairment assessed by the HSCL-11 must fall below the boundary. To reduce the measurement error, symptom improvement must be at least reliable in relation to the impairment level the first time the boundary was surpassed. A detailed description of the failure boundary calculation can be found in [24]. The clinical problem-solving tools are divided into the following domains: (1) risk/suicidality, (2) motivation/treatment goals, (3) therapeutic alliance, (4) social support and critical life events, and (5) emotion regulation/self-regulation.

The TTN is implemented in an online portal, in which therapists can track their patients’ change session-by-session on the HSCL-11 in relation to the expected change trajectory and dynamic failure boundary. When the failure boundary is crossed, and thus, the patient is identified as a not-on-track (NOT) patient, the case is flagged orange. If a patient is NOT and also exceeds a clinical cut-off on a specific domain, the relevant domain is flagged orange and the therapist receives access to the corresponding CST domain. If the domain’s cut-off is not crossed, the tool’s signal remains green and the CST remains inaccessible to the therapist for this particular domain. When a patient goes off track, the clinician receives an email alerting him or her of this event. High values on suicide items are fed back to the clinician immediately after the patient has filled in the HSCL-11 by means of a red bar on the touch screen.

**Fig. 2:**
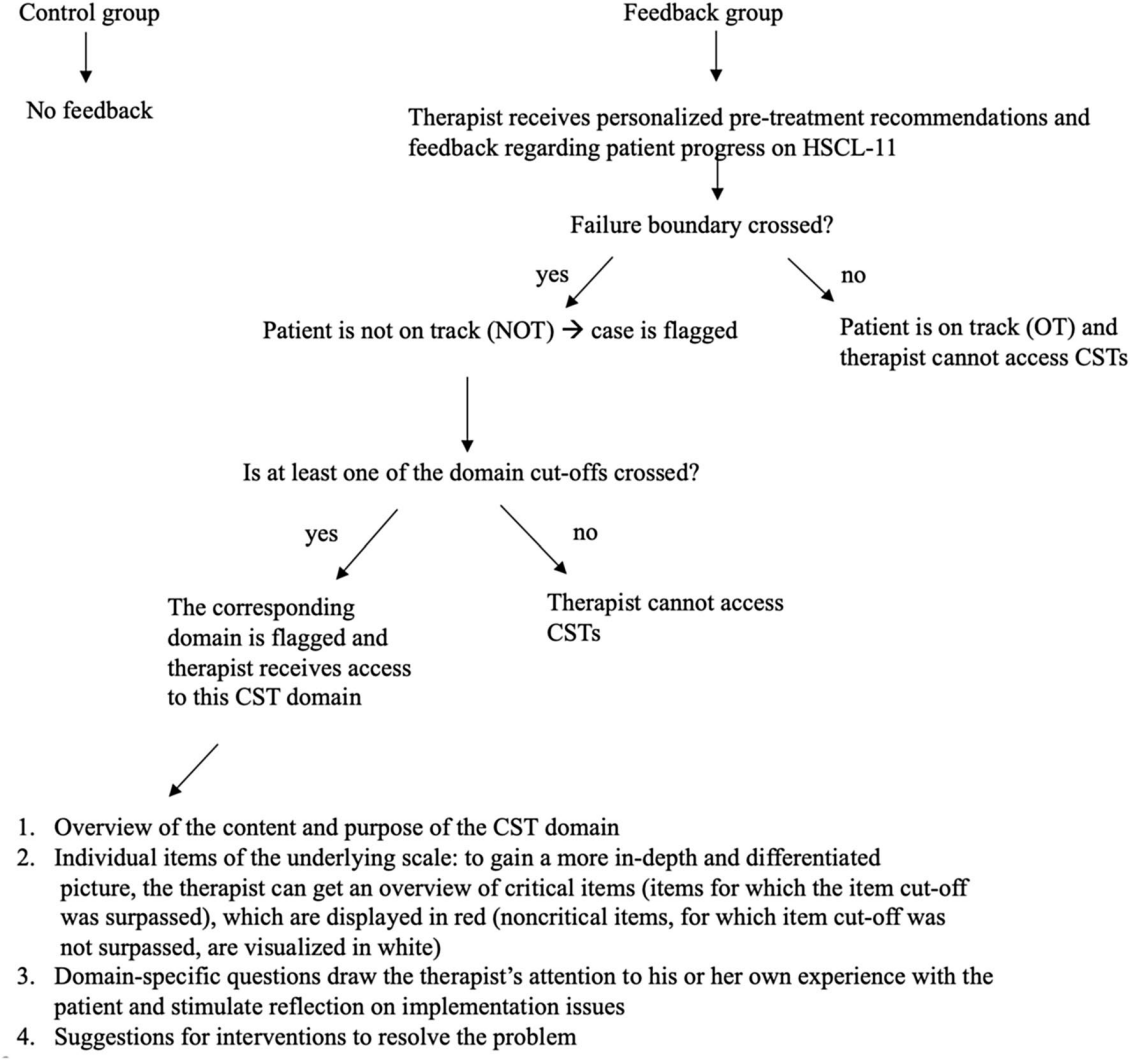
Flow chart of the decision rules within the RCT. *CST* Clinical Support Tool, *HSCL-11* Hopkins Symptom Checklist-11

The TTN displays the following structure for all CST domains: First, a general overview of the content and purpose of the CST domain is provided. Second, therapists are presented with individual items of the underlying scale. Critical items (i.e., the patient surpassed the item cut-off) are marked in red, while non-critical items appear in white. Third, domain-specific questions (e.g., “Which resources could be used for therapy to establish a stable therapeutic alliance?”, “Which concerns might the patient have that should be taken into consideration?”) draw the therapist’s attention to his or her own experience with the patient and stimulate reflection on implementation issues. Fourth, the therapist is provided with suggestions for interventions that can help resolve the problem. Figure 2 summarizes the use of the TTN within the RCT in a flow chart.

### Statistical analysis

To investigate the first research question—(1) Which domains have predictive value for NOT trajectories?—multilevel logistic regression models for dichotomous variables were performed with scores on the scales underlying the CST domains as predictors and OT vs. NOT status as the outcome. Multilevel analyses were performed to account for the hierarchical data structure [36], as advised by the accumulated literature [37–39]. More specifically, sessions (Level 1) were nested within patients (Level 2) and patients within therapists (Level 3). The data were analyzed with the software R version 3.2.0 [40] and the package lme4 [41]. First, an empty model was estimated (without predictors, but with a random intercept, Model 1). The second model contained the random intercept and time (session number) as a fixed effect on the session level. This model was compared to the empty model. The better fitting model (Model 1 vs. Model 2) was further used to identify relevant CST domains. Separate models for each individual domain (risk/suicidality, motivation, therapeutic alliance, life events and social support, emotion regulation/self-regulation) were estimated. Continuous variables were grand-mean centered before entering them into the model. Only predictors that reached significance at a liberal p ≤ 0.10 were included in the final model. The final model (Model 3) was then compared to the better fitting model of the Model 1 versus Model 2 comparison.

To examine the second research question—(2) Do OT and NOT patients differ regarding how often they surpass the domain cut-offs?—NOT and OT patients were compared at session six with regard to signal alerts (crossing the domain’s cut-off) on the different domains by means of Pearson’s chi-square tests. The sixth session was chosen, because it was the first session in which feedback on patient progress status was presented to the therapist.

To examine the third research question—(3) Do OT and NOT patients score differently on the individual items assessing the potential obstacles to a positive treatment outcome?—NOT patients’ sixth session was compared to OT patients’ sixth session regarding the individual items of the ASQ, ASC, and the items forming the risk/suicidality scale. In order to investigate whether the groups differed, independent samples *t*-tests were performed.

In order to create the sum scores of the scales, missing items were replaced by the mean of the respective scale when more than 80% of the items of one scale were available. Otherwise, listwise deletion was applied. For the comparison of individual items, pairwise deletion was applied. As the categorization into OT and NOT only occurred between sessions six and 30 in the RCT and treating therapists received feedback regarding the domains within this time frame only, the analyses focused on these sessions.

## Results

### Sample characteristics

Patients’ mean age was 36.67 (*SD* = 12.95, minimum = 15 and maximum = 77) and the majority of patients were female (*n* = 254, 61.50%). 246 (59.56%) were either married or in a committed relationship. 70 (16.95%) of the patients were unable to work. The most common primary diagnosis was an affective disorder (*n* = 194, 46.97%), followed by an anxiety disorder (*n* = 51, 12.35%). Further diagnoses were posttraumatic stress disorder (*n* = 44, 10.65%), adjustment disorder (*n* = 44, 10.65%), somatoform disorder (*n* = 15, 3.63%), obsessive compulsive disorder (*n* = 14, 3.39%), and eating disorder (*n* = 12, 2.91%). Criteria for a personality disorder were fulfilled for *n* = 88 (21.31%) patients. On average, patients included in the analysis received 26.80 (*SD* = 14.70) sessions of treatment. An overview of patient characteristics for OT and NOT patients can be found in Table 1. OT and NOT patients only differed regarding the variable treatment length, with NOT patients having longer treatments than OT patients.

**Table 1:** Sociodemographic characteristics of the sample by on-track (OT) and not-on-track (NOT) patients

Characteristics	OT patients (*n* = 273)	NOT patients (*n* = 140)	*p* values	Chi^2^ (df)/Cohen’s *d*
Age [*M* (SD)]	36.62 (12.97)	36.75 (12.99)	.93	0.01
Gender (% female)	61.17	62.14	.85	0.04 (1)
Married/committed relationship (% yes)	62.64	53.57	.08	3.16 (1)
Inability to work (% yes)	15.38	20.00	.24	1.40 (1)
Psychiatric drug (% yes)	32.60	38.57	.23	1.46 (1)
Primary diagnosis			.19	3.37 (2)
Affective disorder (%)	44.69	51.43		
Anxiety disorder (%)	14.29	8.57		
Other (%)	41.02	40.00		
Personality disorder (% yes)	20.88	22.14	.77	0.09 (1)
Treatment length (in sessions) [*M* (SD)]	24.09 (14.25)	32.07 (14.17)	** < .001**	**0.56**

*Note* *n* = 413. *p* values were based on *t*-tests for age and treatment length. Cohen’s *d* were calculated using means, standard deviations, and *N*s. Chi^2^ tests were performed for all other variables and the respective Chi^2^ values and degrees of freedom are provided

### Identifying predictors of crossing the failure boundary

Comparing the empty model with the model including time (sessions) as a fixed effect yielded a better fit for the latter [Model 1: Akaike information criterion (AIC): 2982.1, Bayesian information criterion (BIC): 3002.4; Model 2: AIC: 2884.2, BIC: 2911.4]. Hence, the second model was used to investigate further relevant predictors of crossing the failure boundary. Separate models examining time (session number) plus the individual CST domains (entered as fixed effects) were calculated in order to identify those domains that predicted going off track. As shown in Table 2, risk/suicidality, motivation, life events, and social support reached a liberal significance level of *p* ≤ .10 when examined individually and were therefore included in the final model (Model 3). Comparing Model 2 with Model 3 yielded a better fit for Model 3 (AIC: 2671.0, BIC: 2725.3). Besides time (session number), risk/suicidality, motivation, and life events remained significant predictors (*p* ≤ .05) of crossing the failure boundary (see Table 3).

**Table 2:** Fixed effects of crossing the failure boundary in the separate models

	Estimate	Std. error	*z* value	Pro. ( >|*z*|)
Intercept	− 5.68	0.44	− 13.01	**< .001**
Time (session)	0.09	0.01	9.57	**< . 001**
Risk/suicidality	1.79	0.14	12.65	**< .001**
Intercept	− 5.57	0.49	− 11.43	**< . 001**
Time (session)	0.09	0.01	10.14	**< . 001**
Motivation	− 0.13	0.02	− 5.80	**< . 001**
Intercept	− 5.55	0.50	− 11.14	** < . 001**
Time (session)	0.08	0.01	9.50	**< .001**
Therapeutic alliance	− 0.02	0.02	− 0.98	.328
Intercept	− 5.56	0.43	− 12.31	**< . 001**
Time (session)	0. 09	0.01	10.23	**< .001**
Life events	− 0.07	0.01	− 5.50	**< .001**
Intercept	− 5.41	0.46	− 11.66	**< . 001**
Time (session)	0.09	0.01	9.97	**< .001**
Social support	− 0.05	0.01	− 3.72	**< .001**
Intercept	− 5.52	0.49	− 11.21	**< . 001**
Time (session)	0.08	0.01	9.72	**< .001**
Emotion regulation	− 0.01	0.01	−1.18	.239

*Note* Models are based on 6.544 sessions, 413 therapists, and 65 therapists; dummy coded variable 1 = not-on-track and 0 = on-track as dependent variables, for risk/suicidality lower scores indicate higher functioning, for motivation, therapeutic alliance, life events, social support, higher scores indicate higher functioning. Therapist effects (level 3) were estimated to be zero for all models. Differences between patients on level two (patients within therapists) accounted for 79.5–82.7% of the total variance in outcome

**Table 3:** Fixed effects in the final model examining dimensions that predict crossing the failure boundary

	Estimate	Std. error	*z* value	Pro. ( >|*z*|)
Intercept	− 5.67	0.42	− 13.60	**< .001**
Time (session)	0.10	0.01	10.22	**< .001**
Risk/suicidality	1.63	0.14	11.33	**< .001**
Motivation	− 0.09	0.02	− 3.62	**< .001**
Life events	− 0.04	0.01	− 3.05	**.002**
Social support	− 0.02	0.01	− 1.51	.132

*Note n* = 413; dummy coded variable 1 = not-on-track and 0 = on-track as dependent variables, for risk/suicidality lower scores indicate higher functioning, for motivation, therapeutic alliance, life events, social support, higher scores indicate higher functioning

### Comparing the number of cut-off crossings across OT and NOT patients

Overall, NOT patients crossed at least one of the domain cut-offs significantly (*χ*^*2*^ = 17.33, *p* < .001) more often (*n* = 104, 74.29%) than OT patients (*n* = 145, 53.11%). For NOT patients, the cut-off was crossed significantly more often regarding the following domains: risk/suicidality, life events, and social support. For both OT and NOT patients, the emotion regulation domain’s cut-off was most commonly exceeded. Individual results displaying the comparison between OT and NOT patients are presented in Table 4.

**Table 4:** Number of cut-off crossings across the different domains at session 6 for on-track (OT) and not-on-track (NOT) patients and Pearson chi-square tests comparing both patient types

	OT patients (*n* = 273) *n* cut-off crossed (%)	NOT patients (*n* = 140) *n* cut-off crossed (%)	*χ*^2^	df	*p*
Risk/Suicidality	73 (26.74)	54 (38.57)	6.08	1	**.014**
Motivation	11 (4.03)	7 (5.00)	.21	1	.647
Therapeutic alliance	7 (2.56)	9 (6.43)	3.71	1	.054
Life events	14 (5.13)	20 (14.29)	10.27	1	**.001**
Social support	24 (8.79)	25 (17.86)	7.27	1	**.007**
Emotion regulation	96 (35.16)	63 (45.00)	3.78	1	.052

Significant results (*p* < .05) are marked in bold

*Note n* = 413

### Comparing individual items across groups

Independent samples *t*-tests indicated a significant difference between OT and NOT patients on 19 items that are used in the TTN at session six. OT patients showed significantly higher scores regarding four ASQ items, indicating that they were better able to tolerate (other people noticing them) being upset and adjust their bad mood more quickly and easily. Further, one of the ASQ concealing items differed significantly, indicating that NOT patients were less able to control their emotions than OT patients. Moreover, 12 ASC items differed significantly between OT and NOT patients. OT patients had higher values than NOT patients on one of the alliance items, indicating a more trustful alliance between patient and therapist. OT patients scored significantly higher on three social support items, suggesting that they had a better social support network. Also, OT patients scored higher on eight of the life events items than NOT patients, which indicates that they experienced less stressful and critical life events. Both suicidality items (OQ-30 and the HSCL-11) were significantly different for OT and NOT patients, signaling higher suicidality for NOT patients.

Descriptively, for most items, a higher percentage of NOT patients surpassed the item cut-off than OT patients. This suggests that NOT patients tend to have more critical items than OT patients. In comparison to the other scales, the ASC alliance items’ and most of the ASC motivation items’ cut-offs (except*: I am not really sure what to work on in therapy.[ASC #25])* were rarely crossed (7% or less) for both groups, suggesting that patients usually do not report problems on these domains, regardless of OT or NOT status. Some of the other scales’ items, however (*I can get out of a bad mood very quickly*.[ASQ #12], *I know exactly what to do to get myself into a better mood.*[#16], *I can get into a better mood quite easily.*[ASQ #19], *I had support from social groups (like church, school, AA, clubs, *etc*.)[*ASC #19], *I felt connected to a higher power.*[ASC #21], *I shrank from facing a crisis or difficulty.*[ASC #38].), were marked critical very frequently (for 50% or more patients in at least one of the two groups), indicating that many patients displayed difficulties regarding these aspects. A descriptive overview (means and standard error) of the individual items in the two separate groups and the percentage of item cut-off crossings is displayed in online resource 3.

## Discussion

This study aimed to extend knowledge on routine outcome monitoring by comparing patients at risk of treatment failure to patients, whose treatment progress is as expected. We examined whether OT and NOT patients differ with regard to certain factors that have been related to change in the literature and can be regarded as obstacles to a positive treatment outcome. In particular, this study sought to examine whether the domains risk/suicidality, therapeutic alliance, therapy motivation, social support, life events, and emotion regulation have predictive value for NOT trajectories (1st research question). Further, we investigated whether OT and NOT patients differ regarding the frequency of surpassing the domains’ cut-offs (2nd research question) and we also examined the item level to find out whether OT and NOT patients score differently on the individual items assessing these domains (3rd research question).

Overall, the results provide support for the validity of the selected domains’ application. Looking at the predictive value of the individual domains (1st research question), we found that session number, suicidality, therapy motivation, and the occurrence of life events seemed to be predictive of deteriorating in the following sessions. Neither social support, therapeutic alliance, nor emotion regulation predicted going off track in the present study. Thus, in contrast to previous studies [15, 16], social support did not stand out as one of the most important factors of change.

The finding that a higher session number was associated with later deterioration is in line with research investigating sudden losses in psychotherapy (sudden, substantial increases in symptom distress between two consecutive sessions, i.e., sudden deterioration). While sudden gains (sudden, substantial decreases in symptom distress between two consecutive sessions, i.e., sudden improvement) occur rather early in therapy, study results have shown that sudden losses tend to occur later in therapy [38, 42].

The finding that suicidality and risk behavior, which are associated with hopelessness and a lack of adaptive regulation strategies, are predictive of symptom worsening makes theoretical sense. Risk behavior such as drinking or substance abuse should be approached in therapy by identifying triggers in the patient’s daily life, for example. Acute suicidality requires the consideration of alternative treatment approaches or settings and should be discussed with the patient and possibly the supervisor in detail. Implementing this domain into the feedback system can help the clinician to identify and evaluate the risk and may provide information on this topic that would otherwise be lacking [22].

In addition, the findings corroborate the relatively old idea that patients’ therapy motivation and expectations are linked to the initiation and maintenance of change in therapy [43, 44]. More recent studies also support this idea [45]) and especially in addiction treatments, resolving ambivalence has become crucial to prevent drop-out and improve outcome [46]. The findings regarding the domain cut-off crossings and the individual items (results regarding the 2nd and 3rd research questions) show that OT and NOT patients do not differ regarding therapy motivation per se, but that a drop in motivation can promote a negative change trajectory. As motivational problems can have varying causes (e.g., lack of goals, lack of distress, primary or secondary gain [46–49]), therapists need to determine the origin of the motivational problem before implementing interventions.

Further, the association between the occurrence of critical life events and later deterioration seems intuitive and fits with past research findings [16]. Patients seem to be confronted with a problem and have difficulties coping (e.g., because they lack resources), resulting in symptom worsening. Receiving a signal alert, the therapist’s job is to consider the circumstances and think about the impact such an event has on the patient, his or her goals, and therapy and whether the treatment plan should be adjusted. Therapists might, however, feel that for some patients it makes more sense to continue according to the treatment plan.

Investigating the second research question, we were able to identify potential obstacles (i.e., at least one of the domain cut-offs was crossed) for most of the NOT patients (approximately 74%), which was significantly different than the OT patients. This is promising as therapists can use this as a guide to adjust their treatment strategy. However, the number of domain cut-off crossings was also high for OT patients (approximately 53%). For the RCT, this is not problematic, as therapists of OT patients do not receive feedback on these domains anyway. However, this finding may call for the adjustment of the domain cut-offs after data assessment in this study is complete. However, this finding could also indicate that making use of these domains can be helpful in the treatment of OT patients. It has to be noted that this finding refers to session six, in which most NOT patients were still on track. The results show that NOT patients have a higher burden regarding these domains even before going off track. NOT patients showed more deficits regarding risk/suicidality, life events, and social support. Although our results do not suggest that social support predicts changes in symptomatology, NOT patients tended to show more problems regarding their social network than OT patients at session six. Even though social support is an extratherapeutic domain, many different techniques can help patients to improve the quantity and quality of their social network [50, 51]. In order to best help the patient, therapists first need to determine the source of the problem (e.g., role overload, difficult circumstances like moving to a different city or relationship break-up, social skills deficits) before deciding which techniques to implement [51–54]. Exploring the critical items underlying the domain may be helpful. Similar to social support, emotion regulation did not seem to have a very high impact on symptomatic change. However, we did find that both OT and NOT patients showed substantial deficits in emotion regulation, as the domain’s cut-off was most commonly crossed irrespective of group membership.

As described above, the item cut-off alert therapists with NOT patients to the items that are particularly critical for that patient. Thus, the information can help the therapist to get a more in-depth and differentiated picture of the problem within the domain. While exploring the third research question, it became apparent that NOT and OT patients especially differed on the items assessing negative life events and suicidality. This suggests that these items are particularly good indicators of NOT patients. Other items that were significantly different between OT and NOT belonged to the emotion regulation and social support domains. Here again, it must be noted that this finding refers to session six in which most NOT patients were still on track. This indicates that NOT patients tend to have more deficits regarding these items even before going off track. None of the significantly differing items belonged to the motivation domain and only one item belonged to the alliance domain. Further, the percentage of cut-off crossings indicated that item cut-offs within these domains are rarely crossed. This could point to ceiling effects within these domains. Although few patients exceeded the domain and item cut-offs in these two areas, the information therapists can gather from feeding back the individual items can be highly relevant for treatment (e.g., the feeling that the therapist disapproves of oneself). This gives therapists the chance to identify specific problems (although rare) quickly and apply suitable interventions.

Discrepancies between these results and past studies [15, 16] might be explained by the fact that the TTN uses a different and dynamic algorithm to determine OT and NOT patients in comparison to other systems. Further, this study is only one of few studies that actually compared OT and NOT patients regarding such domains. This is a result of institutions having varying routines, for instance, only handing out the ASC when a patient has gone off track instead of administering the questionnaire continuously over the course of treatment. While handing out the questionnaire when patients go off track has the advantage of immediately assessing potential obstacles, handing out the questionnaire in regular intervals to all patients allows for comparative analyses.

In summary, our analyses indicate that particularly focusing on the three domains risk/suicidality, motivation, and life events may prove to be an effective way to prevent treatment failure, as these seem to be directly linked to symptom change. However, the three other scales that do not directly differentiate between OT and NOT patients (social support, alliance, and emotion regulation) can also be helpful to direct clinicians’ attention to problems in NOT cases. Much knowledge is still lacking about the factors that influence change and impact implementation. Future research should build on such findings in order to support therapists to recognize patients at risk and provide effective problem resolution strategies. Further, the findings indicate that several individual items might be more important than others. Thus, questionnaires could be shortened in order to be more efficient in clinical practice.

This study is subject to several limitations. Not all feedback systems make use of the ASC and ASQ in order to determine potential obstacles to a positive treatment outcome (for instance, alliance problems; cf [55].). Therefore, findings are less generalizable to these feedback systems. Further, in the study, not all questionnaires were assessed in the same way: the outcome measurement HSCL-11 was assessed via touch screen, while others were assessed via paper/pencil. In both cases, however, therapists received detailed progress feedback via the TTN system. Also, although OT and NOT patients were very similar regarding most demographic variables, it should be noted that they differed regarding treatment length. However, the finding that negatively developing cases have longer treatments has already been reported in other studies [56]. Further, one of the suicidality items is also used to determine whether patients are considered off track or not. This, of course, increases the chance that NOT patients receive more warning signals regarding risk/suicidality and therefore weakens the validity of the corresponding findings in this study. Further, we decided to compare NOT patients’ sixth session with OT patients’ sixth session, which is somewhat arbitrary. It would be interesting to compare a NOT session with an OT session. However, as OT patients do not have a “key session” like NOT patients, because they do not go off track per definition, we opted to compare the sixth session of both groups. As NOT sessions tend to occur more frequently later in therapy, there might also be good arguments for making a different selection, which could be applied in future studies.

Despite these limitations, the current study provides important insights regarding domains that can play a role for NOT trajectories and can help to inform further improvements of decision-support systems in outpatient psychotherapy.

## Electronic supplementary material

Below is the link to the electronic supplementary material.

Supplementary file1 (PDF 348 kb)
