# Supplementary material for: Why do patients go off track? Examining potential influencing factors for being at risk of psychotherapy treatment failure
Source: Qual Life Res. 2020 Oct 21;30(11):3287–98. doi: 10.1007/s11136-020-02664-6 (PMC8528765; doi:10.1007/s11136-020-02664-6)
Supplement: Supplementary file 1 — Supplementary file1 (PDF 348 kb) [file 11136_2020_2664_MOESM1_ESM.pdf]

**Reference:**

Schilling, V. N. L. S., Zimmermann, D., Rubel, J. A., Boyle, K. S., & Lutz, W. (2020). Why do patients go off track? Examining potential influencing factors for being at risk of psychotherapy treatment failure. *Quality of Life Research*.

**Table ES1:** Domains and item cut-offs used within the TTN

| CST domains | Instruments | Domain cut-off: if cut-off is crossed, domain is flagged orange and the therapist is granted access to the corresponding CST domain | Item cut-off: Within the CST domain, critical items (i.e., the patient surpassed the item cut-off) are marked in red, to shed light on the problematic aspects, while non-critical items appear in white |
|---|---|---|---|
| Risk/suicidality | Items from the HSCL-11 and OQ-30 | As soon as any of the items' cut-offs are crossed | HSCL-suicidality: ≥ 2<br>OQ-suicidality and OQ-risk: ≤ 3 |
| Therapeutic alliance | ASC therapeutic alliance | Subscale total ≤ 39 | ≤ 2 |
| Therapy motivation | ASC motivation | Subscale total ≤ 32 | ≤ 2 |
| Social support | ASC social support | Subscale total ≤ 23 | ≤ 2 |
| Critical life events | ASC life events | Subscale total ≤ 23 | ≤ 2 |
| Emotion regulation/self-regulation | ASQ | Subscale total adjusting ≤ 1.70<br><br>Subscale total tolerating ≤ 2.29<br><br>Subscale total concealing ≤ 2.27 | adjusting: ≤ 2<br><br>tolerating: ≤ 2<br><br>concealing: ≤ 2 |

Note: ASC = Assessment for Signal Clients; ASQ = Affective Style Questionnaire; HSCL = Hopkins Symptom Checklist; OQ = Outcome Questionnaire. ASC-therapeutic alliance: items #1- #11; ASC-social support: items #12- #22; ASC-motivation: items # 23- #31; ASC-life events: items #32-#40; ASQ-concealing: items #1, #2, #5, #9, #10, #13, #15, #18, #20; ASQ-tolerating: items #3, #6, #8, #11, #14, #17; ASQ-adjusting: items #4, #7, #12, #16, #19; HSCL-suicidality: item #10; OQ-suicidality: item #05; OQ-risk: item #18.
The following items were recoded: ASC: #6 - #8, #22 - #29, #31 - #40; ASQ: subscale concealing (#1, #2, #5, #9, #10, #13, #15, #18, #20).

**Figure ES1:** The different components of the TTN

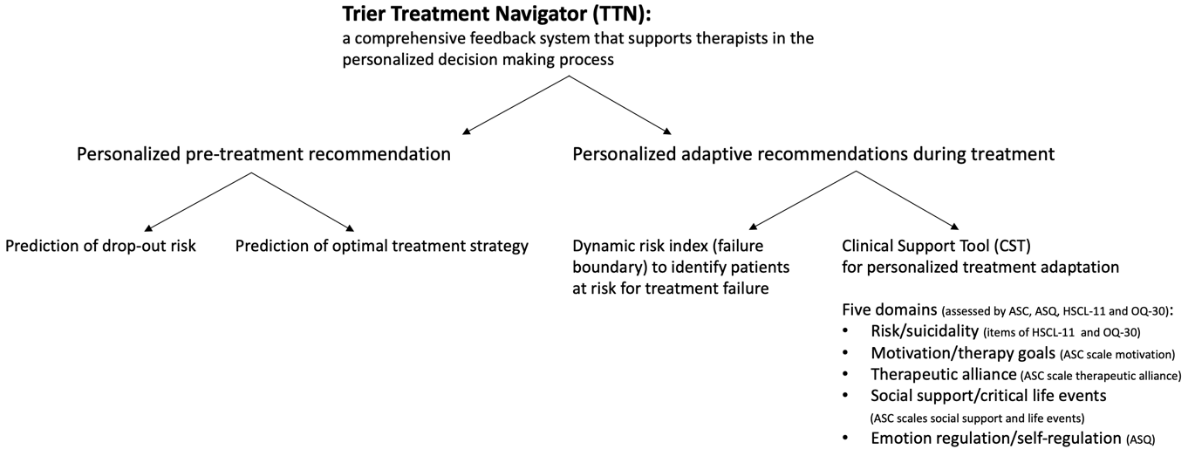

**Table ES2:** Independent samples t-test comparing the mean scores of on-track patients and not-on-track patients (at session 6) regarding the individual items and percentage of surpassing the item cut-off by patient type at session 6.

| **Item** | **OT** | | ***NOT*** | | | | ***Percentage of crossings*** | |
|---|---|---|---|---|---|---|---|---|
| | Mean | *SD* | Mean | *SD* | *t* | *p* | OT | NOT |
| **ASQ** | | | | | | | | |
| 01. People usually can't tell how I am feeling inside. | 2.80 | 1.08 | 2.74 | 1.15 | 0.55 | .584 | 44.0 | 40.7 |
| 02. I have my emotions well under control | 2.87 | 0.98 | 3.11 | 1.06 | -2.19 | **.030** | 33.7 | 28.6 |
| 03. I can tolerate having strong emotions. | 2.95 | 0.99 | 2.76 | 1.09 | 1.64 | .102 | 30.8 | 35.7 |
| 04. I can avoid getting upset by taking a different perspective on things | 2.70 | 1.08 | 2.60 | 1.01 | 0.95 | .342 | 41.0 | 48.6 |
| 05. I often suppress my emotional reactions to things. | 2.91 | 1.12 | 2.76 | 1.16 | 1.26 | .208 | 35.5 | 41.4 |
| 06. It's ok if people see me being upset. | 2.73 | 1.05 | 2.50 | 1.07 | 1.98 | **.049** | 40.7 | 47.9 |
| 07. I can calm down very quickly | 2.70 | 1.07 | 2.51 | 1.03 | 1.66 | .098 | 38.8 | 43.6 |
| 08. I am able to let go of my feelings | 2.64 | 1.08 | 2.70 | 1.16 | -0.51 | .608 | 44.7 | 45.0 |
| 09. I am good at hiding my feelings. | 2.77 | 1.19 | 2.89 | 3.26 | -0.90 | .367 | 43.6 | 34.3 |
| 10. People usually can't tell when I am upset. | 3.26 | 1.14 | 3.22 | 1.15 | 0.36 | .720 | 24.2 | 22.1 |
| 11 It's ok to feel negative emotions at times | 3.37 | 1.03 | 3.28 | 1.07 | 0.83 | .410 | 18.3 | 23.6 |
| 12. I can get out of a bad mood very quickly. | 2.50 | 1.08 | 2.10 | 1.04 | 3.54 | **<.001** | 45.1 | 59.3 |
| 13. People usually can't tell when I am sad. | 2.98 | 1.18 | 3.02 | 1.16 | -0.31 | .759 | 35.5 | 30.7 |
| 14. I can tolerate being upset. | 3.09 | 1.02 | 2.87 | 1.04 | 1.97 | **.050** | 25.3 | 33.6 |
| 15. I can act in a way that people don't see me being upset | 2.99 | 1.16 | 2.95 | 1.22 | 0.35 | .724 | 34.4 | 35.0 |
| 16. I know exactly what to do to get myself into a better mood. | 2.57 | 1.02 | 2.37 | 0.93 | 1.95 | .052 | 46.5 | 57.1 |
| 17. There is nothing wrong with feeling very emotional | 3.30 | 1.00 | 3.10 | 1.16 | 1.69 | .093 | 19.8 | 27.1 |
| 18. I could easily fake emotions. | 3.61 | 1.89 | 3.70 | 1.14 | -0.73 | .456 | 19.4 | 14.3 |
| 19. I can get into a better mood quite easily. | 2.38 | 1.02 | 2.08 | 0.97 | 2.84 | **.005** | 53.5 | 64.3 |
| 20. I can hide my anger well if I have to. | 2.85 | 1.18 | 2.93 | 1.25 | -0.62 | .535 | 41.0 | 36.4 |
| **ASC** | | | | | | | | |
| 01. I felt cared for and respected as a person. | 4.46 | 0.70 | 4.39 | 0.84 | 0.73 | .466 | 1.5 | 3.6 |
| 02. I felt my therapist understood me. | 4.46 | 0.62 | 4.43 | 0.73 | 0.40 | .687 | 0.4 | 0.7 |
| 03. I thought the suggestions my therapist made were useful. | 4.05 | 0.84 | 4.08 | 0.91 | -0.32 | .748 | 1.1 | 2.1 |
| 04. I felt like I could trust my therapist completely. | 4.45 | 0.73 | 4.23 | 0.78 | 2.69 | **.008** | 0.7 | 1.4 |
| 05. I was willing to share my innermost thoughts with my therapist. | 4.11 | 0.90 | 4.10 | 0.86 | 0.09 | .932 | 4.4 | 5.0 |

| | | | | | | | | |
|---|---|---|---|---|---|---|---|---|
| 06. I felt there was a breakdown in the relationship with my therapist. | 4.89 | 0.42 | 4.88 | 0.49 | 0.21 | .834 | 0.4 | 0.7 |
| 07. I felt like my therapist disapproved of me. | 4.90 | 0.48 | 4.90 | 0.48 | 0.07 | .946 | 1.1 | 0.7 |
| 08. At times, the tone of my therapist's voice seemed critical or impatient. | 4.89 | 0.46 | 4.82 | 0.62 | 1.24 | .215 | 0.7 | 1.4 |
| 09. My therapist seemed to be glad to see me. | 3.88 | 0.81 | 3.84 | 0.83 | 0.46 | .643 | 1.1 | 2.1 |
| 10. My therapist and I seemed to work well together to accomplish what I want. | 4.28 | 0.66 | 4.20 | 0.73 | 0.97 | .332 | 0.0 | 0.7 |
| 11. My therapist and I had a similar understanding of my problems. | 3.99 | 0.73 | 3.92 | 0.83 | 0.80 | .426 | 1.5 | 0.7 |
| 12. I could count on my friendships when things went wrong. | 3.56 | 1.19 | 3.21 | 1.27 | 2.63 | **.009** | 15.8 | 27.1 |
| 13. I could talk about problems with my friends. | 3.27 | 1.27 | 3.18 | 1.37 | 0.61 | .544 | 24.5 | 30.0 |
| 14. I could talk about problems with my family. | 3.17 | 1.33 | 2.91 | 1.33 | 1.82 | .070 | 30.4 | 39.3 |
| 15. I got the emotional help and support I needed from someone in my family. | 3.10 | 1.37 | 2.92 | 1.41 | 1.15 | .253 | 30.0 | 37.9 |
| 16. There was a special person who was around when I was in need. | 3.92 | 1.27 | 3.53 | 1.33 | 2.78 | **.006** | 15.4 | 22.9 |
| 17. There was a special person with whom I could share my joys and sorrows. | 3.80 | 1.28 | 3.56 | 1.30 | 1.70 | .091 | 15.8 | 22.1 |
| 18. I could get material support if needed (like: money, food, transportation, child care, tools, repairs, health care, legal advice, etc.) | 3.03 | 1.43 | 3.00 | 1.56 | 0.21 | .832 | 29.3 | 32.9 |
| 19. I had support from social groups (like: church, school, AA, clubs, etc.) | 1.86 | 1.23 | 1.84 | 1.28 | 0.16 | .875 | 64.1 | 65.0 |
| 20. I felt accepted by someone other than my therapist. | 4.00 | 1.05 | 3.85 | 1.15 | 1.26 | .209 | 7.0 | 12.1 |
| 21. I felt connected to a higher power. | 1.41 | 0.89 | 1.57 | 1.06 | -1.49 | .139 | 82.1 | 76.4 |
| 22. Some subjects were so sensitive I couldn't talk with anyone about them. | 3.69 | 1.35 | 3.25 | 1.38 | 2.97 | **.003** | 19.4 | 29.3 |
| 23. I wonder what I am doing in therapy; actually I find it boring. | 4.83 | 0.44 | 4.82 | 0.54 | 0.34 | .734 | 0.4 | 1.4 |
| 24. Honestly, I really don't understand what I can get from therapy. | 4.79 | 0.57 | 4.76 | 0.63 | 0.42 | .674 | 1.1 | 1.4 |
| 25. I am not really sure what to work on in therapy. | 4.16 | 1.18 | 4.21 | 1.11 | -0.43 | .670 | 13.2 | 11.4 |
| 26. I had thoughts about quitting therapy; it's just not for me. | 4.86 | 0.47 | 4.90 | 0.41 | -0.84 | .401 | 0.7 | 0.7 |
| 27. I don't think therapy will help me feel any better. | 4.59 | 0.84 | 4.48 | 0.88 | 1.20 | .233 | 3.7 | 4.3 |
| 28. I have no desire to work out my problems. | 4.89 | 0.38 | 4.83 | 0.60 | 1.18 | .238 | 0.4 | 2.9 |
| 29. Although I am currently unhappy with life, there is nothing I can do about it now. | 4.36 | 0.99 | 4.19 | 1.06 | 1.55 | .123 | 7.0 | 7.1 |
| 30. Through therapy I am taking more responsibility for changing my life. | 3.98 | 0.95 | 4.06 | 0.97 | -0.74 | .461 | 6.2 | 5.0 |
| 31. I am in therapy because someone is requiring it of me. | 4.76 | 0.71 | 4.78 | 0.75 | -0.30 | .763 | 2.9 | 2.9 |
| 32. I had an interaction with another person that I found upsetting. | 3.33 | 1.51 | 3.02 | 1.47 | 1.20 | **.047** | 37.0 | 40.7 |
| 33. I felt rejected or betrayed by someone. | 3.81 | 1.39 | 3.18 | 1.48 | 4.06 | **<.001** | 20.9 | 37.1 |
| 34. I made a mistake that I can't undo. | 4.53 | 0.93 | 4.23 | 1.25 | 2.43 | **.016** | 4.8 | 13.6 |
| 35. I received bad news that was difficult for me. | 3.94 | 1.40 | 3.60 | 1.50 | 2.20 | **.028** | 21.6 | 27.1 |
| 36. I lost a person I was close to. | 4.77 | 0.78 | 4.56 | 1.04 | 1.95 | .052 | 3.7 | 7.9 |
| 37. There was trouble at home, work, or school. | 4.05 | 1.26 | 3.49 | 1.46 | 3.73 | **<.001** | 14.7 | 32.1 |

| | | | | | | | | |
|---|---|---|---|---|---|---|---|---|
| 38. I had health problems (such as physical pain). | 3.40 | 1.56 | 2.73 | 1.48 | 4.18 | **<.001** | 35.9 | 50.0 |
| 39. I shrank from facing a crisis or difficulty. | 3.79 | 1.24 | 3.29 | 1.24 | 3.79 | **<.001** | 19.0 | 28.6 |
| 40. I had difficulty adjusting to an occurrence in my life. | 3.67 | 1.32 | 3.08 | 1.40 | 3.97 | **<.001** | 19.0 | 36.4 |
| **OQ-30** | | | | | | | | |
| 05. I have thoughts of ending my life. | 0.36 | 0.81 | 0.65 | 0.96 | -3.03 | **.003** | 4.4 | 5.0 |
| 18. I feel annoyed by people who criticize my drinking (or drug use). | 0.17 | 0.53 | 0.18 | 0.56 | -1.07 | .915 | 1.5 | 1.4 |
| **HSCL-11** | | | | | | | | |
| 10. In the past seven days, how much were you distressed by thoughts of ending your life. | 1.33 | 0.62 | 1.49 | 0.72 | -2.25 | **.025** | 24.9 | 36.4 |

Note: *n* = 413; significant results ($p < .05$) are marked in bold; Abbreviations: ASC =Assessment for Signal Clients (high values indicate high functioning); ASQ = Affective Style Questionnaire (high values indicate high functioning); HSCL = Hopkins Symptom Checklist (low values indicate low suicidality); NOT = not-on-track; OT = on-track; OQ = Outcome Questionnaire (low values indicate low suicidality/risk).
